# Impacts of right ventricular trabeculae and papillary muscles on volumes and function assessed by cardiovascular magnetic resonance using a novel software: semi-automatic threshold-based segmentation algorithm

**DOI:** 10.1186/1532-429X-17-S1-P73

**Published:** 2015-02-03

**Authors:** Akio Inage, Naokazu Mizuno

**Affiliations:** 1 Department of Radiology, Sakakibara Heart Institute, Fuchu, Japan

## Background

The objective of this study was to assess the impact of right ventricular (RV) trabeculae and papillary muscles on measured volumes and function assessed by cardiovascular magnetic resonance (CMR) using a novel semi-automatic segmentation algorithm in patients with right heart lesion of the congenital heart diseases. The new algorithm was based on the signal intensity distribution of MR images, and excludes trabeculae and papillary muscles from the blood pool, while the manual approach includes these objects in the blood pool.

## Methods

We measured RV end-diastolic volume (RVEDV), end-systolic volume (RVESV), stroke volume (RVSV) and ejection fraction (RVEF) by CMR using standard methods of manual contour tracing and semi-automatic methods. We measured net pulmonary artery forward flow volume (PAFV) as RVSV using phase contrast (PC) MR. Also, we measured RVEDV, RVESV, RVSV and RVEF using ventriculographies including trabeculae and papillary muscles. The new algorithm was performed using the Medis novel software.

## Results

There was a total of 50 cases and the mean age was 22 +/- 18 years. By excluding the trabeculae and papillary muscle in the RV blood volume, the measured RVEDV decreased by 31 % (from 175 +/- 64 to 120 +/- 66 ml/m^2^, p < 0.005), RVESV by 33 % (from 108 +/- 67 to 72 +/- 47 ml/m^2^, p < 0.005), RVSV by 25 % (from 60 +/- 28 to 45 +/- 29 ml/m^2^, p < 0.005) and the RVEF slightly increased by 7 % (from 40 +/- 15 to 43 +/- 19 %, p = 0.01). RVSV measured by PAFV using PC (42 +/- 24 ml/m^2^) was more related with value measured by semi-automatic methods (mean difference = 3.8 ml/m^2^, p = 0.37) rather than the standard methods (mean difference = 18.3 ml/m^2^, p < 0.005) on MRI. In 32 cases, RVSV measured by ventriculographies (53 +/- 18 ml/m^2^) was more related with value measured by standard methods (mean difference = 2.1 ml/m^2^, p = 0.4) rather than the semi-automatic methods (mean difference = 13.8 ml/m^2^, p < 0.005) on MRI.

## Conclusions

The measured RV volumes and function significantly changed by excluding trabeculae and papillary muscle. The new semi-automatic threshold-based segmentation software can reliably exclude trabeculae and papillary muscles from the RV blood volume.

## Funding

None.

**Figure 1:**
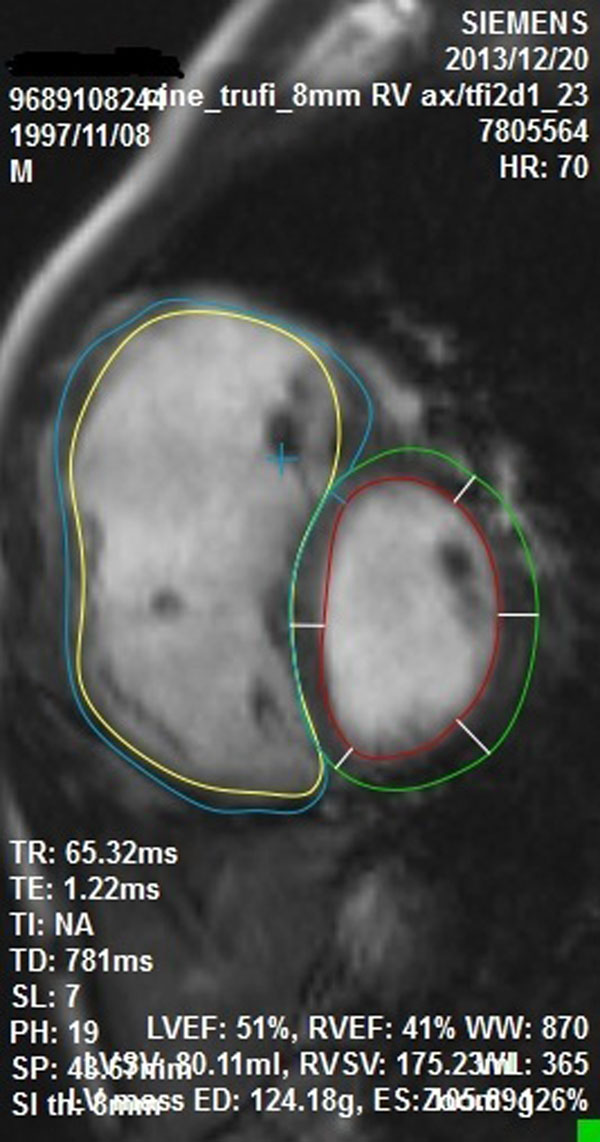
Manual contour tracing.

**Figure 2:**
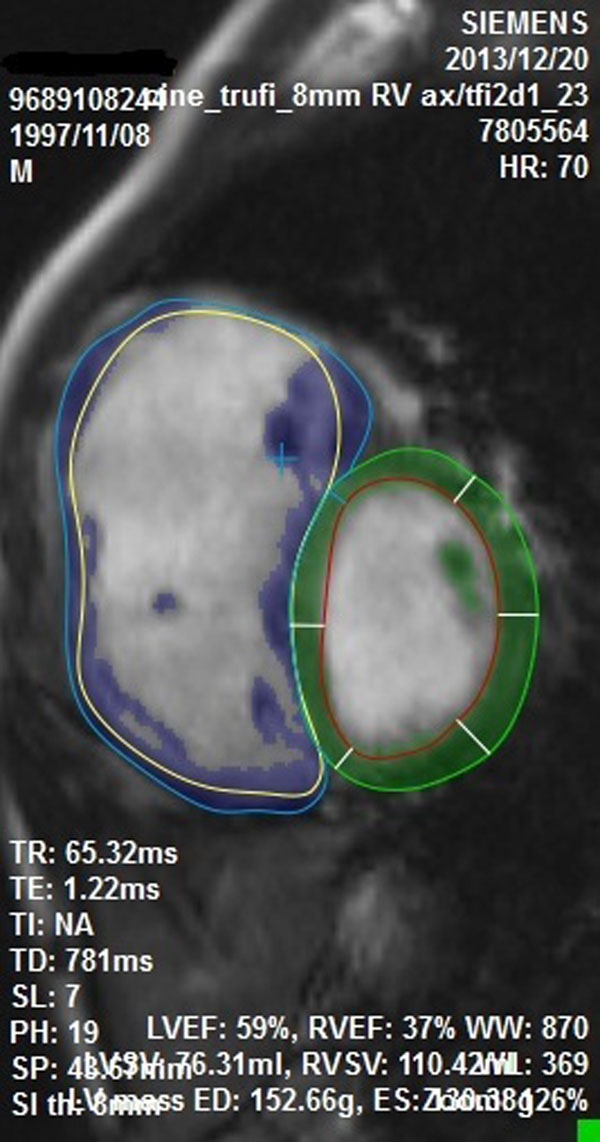
Semi-automatic segmentation algorithm.

